# Maximizing the performance of heat stressed broilers by optimizing starch-to-lipid ratios, digestible amino acid, and metabolizable energy during the finisher phase

**DOI:** 10.1016/j.psj.2024.104729

**Published:** 2024-12-24

**Authors:** Dilshaan Duhra, Denise Beaulieu, Tory Shynkaruk, Juliano C. de Paula Dorigam, Rose Whelan, Karen Schwean-Lardner

**Affiliations:** a College of Agriculture and Bioresources, University of Saskatchewan, 51 Campus Drive, Saskatoon, Saskatchewan S7N 5A8, Canada; b Evonik Operations GmbH, Hanau-Wolfgang, Essen 63457, Germany

**Keywords:** Amino acid, Broiler, Growth performance, Meat yield, Energy

## Abstract

This study investigated the effects and interactions among diets formulated to have high starch-to-lipid ratios (S:L), amino acid density [indicated as % digestible lysine (DigLys)], and AME on growth performance and carcass characteristics of heat stressed broilers. A {3,3} simplex lattice design was used to assess relative effects and generate predictive models. Three basal finisher diets were formulated to have the highest S:L ratio (Basal A; 20:1), DigLys (Basal B; 1.30 %), or AME (Basal C; 3300 kcal/kg). These diets were blended at levels of 0.00, 0.33, 0.67, or 1.00 to produce 10 finisher diets. The mixtures allowed varying S:L ratios (4:1 to 20:1), DigLys (0.80 to 1.30 %), and AME (2800 to 3300 kcal/kg) content of diets. sex-separated (*n* = 6,864) Ross 708 broiler chicks were placed in separate rooms (5 male and 4 female) with a pen stocking density of 31 kg/m^2^. Sex-specific starter and grower diets were fed until d 21. The rooms were maintained at 21°C during d 21 to 27. From d 27 to 32, the birds were subjected to cyclical heat stress, with 12 h of 31°C followed by 12 h of 21°C, with a minimum RH of 50 %. BW and feed residual weights were measured on d 21, 27, and 32, then used to calculate BW gain (BWG) and feed-to-gain ratios (F:G). On d 33, 20 birds per treatment per sex were slaughtered to determine carcass characteristics. Under these conditions (d 21 to 32), maximum male BWG of 926 g was estimated to occur when fed a diet comprised of 42.2 % Basal B and 57.8 % Basal C with a S:L ratio of 4:1, AME of 3089 kcal/kg, and 1.01 % DigLys. Diet did not influence female BWG during heat stress. Although a practical recommendation was not possible for optimal breast meat yield (% live weight) and F:G ratios, the results, indicated that increasing DigLys would improve these parameters under heat stress.

## Introduction

Rising global temperatures are challenging poultry producers to find cost effective strategies to alleviate negative effects resulting from heat stress. As birds are unable to sweat, they are primarily reliant on panting for thermoregulation, which is less effective when exposed to the combination of high temperature and RH (Nawab et al., 2018). Birds experience heat stress when the environmental conditions exceed their tolerance level, utilizing both behavioral and physiological mechanisms to lower their body temperature. Heat stressed birds will exhibit reduced activity and, limit their feed intake which reduces metabolic heat production, and growth rate, while increasing mortality, and feed-to-gain (**F:G**) ratios (Goo et al., 2019; Awad et al., 2020).

Broiler chickens are particularly susceptible to heat stress as they have been selectively bred for fast growth, resulting in increased metabolic activity. Studies have reported that heat stress in broilers leads to significant reductions in feed intake and growth (Goo et al., 2019; Awad et al., 2020). Heat stress has also been reported to reduce meat yield, increase fat deposition (Teyssier et al., 2022), and reduce meat quality in broilers (Zaboli et al., 2018).

Studies have proposed measures to ameliorate the negative impact of heat stress such as reducing dietary CP with supplementation of limiting amino acids to reduce heat increment (Attia et al., 2020) or improving the amino acid to energy ratios by increasing amino acid density to 110–120 % of recommended levels (Maharjan et al., 2020). Studies have also found that increasing dietary AME in the form of lipids improved BW gain (**BWG**), F:G ratios, and protein utilization in heat stressed broilers. However, fat deposition increased as well (Ghazalah et al., 2008; Attia and Hassan, 2017). Starch-to-lipid (**S:L**) ratios may also be an important factor as low AME diets with high starch-to-lipid ratios resulted in increased fat deposition in broilers (Khoddami et al., 2018). However, most studies have focused on improving heat stressed broiler performance by modifying dietary recommendations for birds reared under thermoneutral conditions. As such, the objective of this study was to determine the optimal mixture of three basal diets formulated to have high S:L, digestible amino acid content (**DigLys**), and AME that maximize growth performance of broilers exposed to cyclic heat stress from d 27 to 32. The diets were formulated to exceed requirements to allow maximum or minimum responses in target metrics such as BWG and F:G ratios. The high S:L diet was chosen to assess if there were differences between starch or lipids as energy sources on performance. These diets were then used to develop a recommendation for diet composition with regards to DigLys, AME, starch, and lipids as a starting point for further development.

## Materials and methods

The experimental protocol for this trial was approved by the University of Saskatchewan's Animal Care Committee (AUP 20210085) and was performed under the recommendations as specified in the Guide on the Care and Use of Experimental Animals by of the Canadian Council on Animal Care, 2009.

### Simplex lattice design

A simplex lattice, a type of mixture design, was used for this experiment. Mixture designs were developed as a derivative of response surface models by Scheffé (1958) where the factors are components of a mixture in which the sum of the proportions equals one. The goal of the design is to optimize the target response to achieve a minimum, maximum, or a specific value as well as determining the relative effects of the components to the others. The components therefore, are bound in value between zero and one. If q components are present in a mixture, such as the three basal finisher diets used during this trial, q-1 components are required to determine the effect of the last component constraining the model to a two-dimensional space resulting in a design of an equilateral triangle (simplex lattice) where:

(1) The vertices represent a single basal diet.
(2) Points along the perimeter consist of blends of two basal diets.
(3) A central point consists of equal proportions of all three basal diets.

As the constraint of this model is that all components must sum to one, model coefficients are not individually determined. Because of this, a factorial design would not yield the results needed to analyze these mixtures because the components are dependent on each other.

The model fitted is

*Y* = *β*a*x*a + *β*b*x*b + *β*c*x*c + *β*ab*x*a*x*b + *β*ac*x*a*x*c+ *β*bc*x*b*x*c

Where *β*a, *β*b, and *β*c are the individual effects of each basal diet. The values for *x*a, *x*b, and *x*c total to one and are the proportions of the basal diets A, B, and C. *β*ab, *β*ac, and *β*bc are the effects of each interaction for binary blends of basal diets A, B, and C which are only included if the interaction is determined to influence the target metric.

### Diets

To maximize growth, male and female broilers were provided with sex-specific starter (d 0-10) and grower diets (d 10-21) (Table 1) formulated according to AMINOChick ® 3.0 recommendations (Lemme, 2021). The experimental diets in the finisher phase were based on the combination of three basal diets (Table 2) formulated to have a high S:L ratio (Basal A; 20:1), DigLys (Basal B; 1.30 %), or AME (Basal C; 3300 kcal/kg). These diets were formulated to be extremes in composition to allow detection of minimums or maximums in performance. This resulted in Basal B having a higher formulated CP content to meet amino acid requirements. The model analyzed effects of the whole diet, so any effects associated with the high CP would be accounted for as part of the generated models. The basal diets were blended at equally spaced levels of 0.00, 0.33, 0.67, and 1.00 to produce 10 finisher diets (Tables 3, 4). The mixtures allowed varying S:L ratios (4:1 to 20:1), DigLys (0.80 to 1.30 %), and AME (2,800 to 3300 kcal/kg). Ingredients used in the feed formulations were analyzed by near-infrared spectroscopy using AMINONIR® Advanced (Evonik Operations Gmbh, Hanau-Wolfgang, Essen, Germany). All finisher diets contained 0.5 % diatomaceous earth (EP Minerals, Reno, NV), which was used as a marker to measure nutrient retention.

**Table 1:** Starter and grower diet formulations (% as fed).

Ingredient (%)	Starter diets (0-10 days)		Grower diets (10-21 days)	
	Male	Female	Male	Female
Corn	50.879	51.415	57.014	58.636
Soybean meal 45 % CP	37.955	37.909	32.601	32.449
Canola oil	3.442	3.474	4.173	4.264
Corn gluten meal	2.889	2.378	1.816	0.287
Dicalcium phosphate	2.127	2.129	1.921	1.926
Limestone	0.861	0.860	0.791	0.788
Salt	0.319	0.319	0.318	0.319
Sodium bicarbonate	0.100	0.101	0.107	0.111
Choline chloride 60	0.101	0.101	0.103	0.103
Premix^1^	0.500	0.500	0.500	0.500
Biolys 60^2^	0.350	0.341	0.280	0.236
DL-methionine	0.326	0.326	0.275	0.274
L-threonine	0.085	0.082	0.064	0.067
L-valine	0.066	0.066	0.037	0.040

1 Premix contains 2,400,000 IU/kg vitamin A, 700,000 IU/kg vitamin D3, 20,000 IU/kg vitamin E, 4000 µg/kg vitamin B12, 50,000 µg/kg biotin, 600 mg/kg menadione, 500 mg/kg thiamine, 1400 mg/kg riboflavin, 6000 mg/kg pantothenic acid, 1000 mg/kg pyridoxine, 14,000 mg/kg niacin, 400 mg/kg folic acid, 12,000 mg/kg iron, 4,000 mg/kg copper, 24,000 mg/kg manganese, 22,000 mg/kg zinc, 500 mg/kg iodine, and 60 mg/kg selenium (DSM Nutritional Products Canada Inc. Ayr, Ontario, Canada).

2 Minimum 60 % l-lysine (Evonik Industries GmbH, Hanau-Wolfgang, Essen, Germany).

**Table 2:** Basal finisher diet formulations (% as fed).

Ingredient (% as fed)	Basal A	Basal B	Basal C
	High S:L ratio	High DigLys	High AME
Corn	41.398	21.752	61.043
Corn starch	22.040	13.748	0.000
Soybean meal 45 % CP	18.592	30.726	16.233
Wheat middlings	6.462	14.381	7.103
Corn gluten meal	1.949	1.821	2.651
Soybean oil	0.000	5.220	7.246
Premix^1^	0.500	0.500	0.500
Dicalcium phosphate	1.857	1.693	1.829
Limestone	0.733	0.728	0.757
Sodium bicarbonate	0.271	0.268	0.295
Salt	0.308	0.309	0.294
Choline chloride 60	0.168	0.109	0.156
Sand	2.897	4.000	0.454
Silica dioxide	1.449	2.000	0.227
Biolys 60^2^	0.347	0.698	0.359
DL-methionine	0.222	0.508	0.168
L-threonine	0.100	0.279	0.070
L-arginine	0.079	0.258	0.067
L-valine	0.071	0.286	0.021
L-isoleucine	0.058	0.220	0.027
Celatom celite^3^	0.500	0.500	0.500

Abbreviations: S:L (starch-to-lipid), DigLys (digestible amino acid content).

1 Premix contains 2,400,000 IU/kg vitamin A, 700,000 IU/kg vitamin D3, 20,000 IU/kg vitamin E, 4000 µg/kg vitamin B12, 50,000 µg/kg biotin, 600 mg/kg menadione, 500 mg/kg thiamine, 1400 mg/kg riboflavin, 6000 mg/kg pantothenic acid, 1000 mg/kg pyridoxine, 14,000 mg/kg niacin, 400 mg/kg folic acid, 12,000 mg/kg iron, 4000 mg/kg copper, 24,000 mg/kg manganese, 22,000 mg/kg zinc, 500 mg/kg iodine, and 60 mg/kg selenium (DSM Nutritional Products Canada Inc. Ayr, Ontario, Canada).

2 Minimum 60 % l-lysine (Evonik Industries GmbH, Hanau-Wolfgang, Essen, Germany).

3 Minimum 89 % diatomaceous earth (EP Minerals, Reno, NV).

**Table 3:** Proportions of the basal diets used to compose the experimental finisher diets and their respective calculated nutritional composition.

Treatments	Proportions of the 3 basal diets (A, B, and C; % as fed)	S:L ratio	DigLys (% as fed)	AME (kcal/kg)
1	0.00, 1.00, 0.00	4.0	1.30	2,800
2	0.00, 0.67, 0.33	4.0	1.13	2,967
3	0.00, 0.33, 0.67	4.0	0.97	3,133
4	0.00, 0.00, 1.00	4.0	0.80	3,300
5	0.33, 0.00, 0.67	5.6	0.80	3,133
6	0.33, 0.33, 0.33	5.9	0.97	2,967
7	0.33, 0.67, 0.00	6.2	1.13	2,800
8	0.67, 0.00, 0.33	9.0	0.8	2,967
9	0.67, 0.33, 0.00	10.2	0.97	2,800
10	1.00, 0.00, 0.00	20.0	0.80	2,800

**Table 4:** Estimated and analyzed nutritional composition of finisher diets.

	1	2	3	4	5	6	7	8	9	10
Estimated nutrient composition (% as fed)										
Dry matter	88.938	88.835	88.731	88.627	88.509	88.613	88.716	88.391	88.495	88.273
AME (kcal/kg)	2800	2967	3133	3300	3133	2967	2800	2967	2800	2800
Starch	29.200	33.067	36.933	40.800	42.733	38.867	35.000	44.667	40.800	46.600
Crude fat	7.300	8.267	9.233	10.200	7.577	6.610	5.643	4.953	3.987	2.330
S:L ratio	4.000	4.000	4.000	4.000	5.640	5.880	6.202	9.017	10.234	20.000
Crude protein	21.100	19.329	17.558	15.787	15.509	17.280	19.051	15.232	17.003	14.954
Calcium	0.790	0.790	0.790	0.790	0.790	0.790	0.790	0.790	0.790	0.790
Available Phosphorus	0.395	0.395	0.395	0.395	0.395	0.395	0.395	0.395	0.395	0.395
Sodium	0.200	0.200	0.200	0.200	0.200	0.200	0.200	0.200	0.200	0.200
Chloride	0.220	0.220	0.220	0.220	0.220	0.220	0.220	0.220	0.220	0.220
Digestible Methionine + Cysteine	0.990	0.863	0.737	0.610	0.610	0.737	0.863	0.610	0.737	0.610
Digestible Methionine	0.756	0.637	0.517	0.398	0.407	0.526	0.645	0.415	0.534	0.424
Digestible Lysine	1.300	1.133	0.967	0.800	0.800	0.967	1.133	0.800	0.967	0.800

Analyzed nutrient composition (% as fed)										
Crude protein	21.31	20.20	17.97	16.31	16.43	17.93	18.96	17.12	18.45	16.37
Methionine + Cysteine	1.112	0.977	0.853	0.690	0.749	0.812	1.003	0.749	0.897	0.761
Methionine	0.790	0.682	0.561	0.409	0.447	0.516	0.700	0.469	0.612	0.499
Lysine	1.44	1.182	1.083	0.924	0.986	1.127	1.321	0.998	1.180	0.967

### Housing

A total of 6864 sex-separated Ross 708 broilers were housed in 9 independent, temperature-controlled rooms (5 male, 4 female rooms), with 12 pens (2.0 × 2.3 m) per room, with an estimated final stocking density of 31 kg/m^2^ (68 females/pen, 60 males/pen; Aviagen, 2019). The lighting program began with 22L:2D, decreasing to 20L:4D by d 2 with a 15-min dawn to dusk system. Light intensity was 40 lux on d 1 of the trial and was gradually reduced to 10 lux by d 9. Initial room temperature was 32°C and decreased to 21 °C by d 21. During the heat stress period (d 27 to 32) mimicking a heat wave which could be observed in North America of approximately 3–6 days (Rastogi et al., 2020; Ji et al., 2022), temperature was maintained at 31 ⁰C for 12 h per d from 8am-8pm with a nighttime temperature of 21 ⁰C (1 h heat up and cool down period for each transition). Humidity was maintained at 50-65 % for the first week with a minimum 50 % humidity for the remainder of the trial. Wood shavings were used as the litter source. Pens contained a tube feeder (36 cm pan diameter d 0–21 and 43 cm diameter until d 32) and 6 nipple drinkers. Birds were placed at 0 d of age and reared in pens until 32 d.

On d 21, all broilers were weighed on a pen basis and switched onto the experimental finisher diets. Ten pens (2.0 × 2.3 m) per room were used for the trial, with 2 additional pens used to provide birds to replace mortalities occurring during the initial 21 d so all study pens began the experimental portion of the trial at a consistent stocking density (60 males or 68 females/pen, 31 kg/m^2^). At d 21, 8 birds per treatment per sex were also housed in two bioassay cages (51 cm wide × 51 cm long x 46 cm high; 4 birds per cage), which allowed for excreta collection to determine nitrogen retention, AME, and dry matter retention while under the effects of heat stress. Birds in these cages were exposed to the same heat stress, lighting, humidity, and temperature conditions as described above.

### Performance parameters

Body weight was measured on a pen basis at d 0, 21, 27, and 32 and values were used to calculate BWG. Residual feed was weighed on d 21, 27, 30, and 32 on a per pen basis, allowing for the calculation of average feed intake/bird (**FI**) for each period. F:G ratios were calculated using BW and FI for each period accounting for mortalities. All culls and mortalities were weighed and recorded. From d 7 the cause of mortality was determined by a trained pathologist at an independent necropsy facility (Prairie Diagnostic Services Inc, Saskatoon, Saskatchewan, Canada).

### Carcass characteristics

Meat yield was measured at the end of the trial on 20 birds/treatment/sex (4 males/pen, 5 females/pen). Live weights were recorded prior to an 8 h feed withdrawal period. Birds were shackled, stunned via an electric knife, and exsanguinated by cutting the carotid arteries and jugular veins. Carcasses were scalded at 62–64 °C (30 s), mechanically plucked, manually eviscerated, chilled in an ice bath for one h, and then placed on ice for a minimum of 16 h. Carcass, *Pectoralis* (*P.*) major and minor, breast skin, wing, whole thigh, whole drum, and remaining back and rack weights were recorded.

### Nutrient retention

Excreta and diets were analyzed for nitrogen (**N**), gross energy (**GE**), and DM, allowing the calculation of N retention, dietary AME, and DM retention. Excreta was collected from d 30 to 32. Clean aluminum trays were placed under all cages to collect uncontaminated excreta. Excreta was stored at −20 ⁰C until analyses. It was then dried at 55 °C and ground through a 1 mm screen. DM was assessed by drying at 135 ⁰C for 2 h (AOAC, 2016). Nitrogen was measured using a Leco auto-analyzer, which combusted the sample with pure oxygen at 850 °C, an aliquot of gas was then passed through a copper catalyst, which converted nitrous oxides to N_2_, N content was determined by measuring thermal conductivity (AOAC, 2016). Gross energy was determined using adiabatic bomb calorimetry (Parr instrument Co., Moline, IL). Acid-insoluble-ash (**AIA**) was measured using a procedure from Vogtmann et al. (1975). N, DM retention, AME, and AMEn were calculated using the following equations respectively:

%N retention = 100*(1 - (%NExcreta / %AIAFeed) * (%AIAExcreta / %NFeed)

%DM retention = 100*(1 - (%DMExcreta / %AIAFeed) * (%AIAExcreta / %DMFeed)

AME = GEFeed – ((GEexcreta * %AIAfeed)/ %AIAexcreta)

AMEn = GEFeed – ((GEexcreta * %AIAfeed)/ %AIAexcreta) – 8.22 * (%Nfeed – (%NExcreta * %AIAfeed / %AIAexcreta))

### Heterophil-to-lymphocyte (H:L) ratio

On d 31, approximately 2 mL of blood were collected from the brachial vein of 2 birds/pen. Slides were prepared using the two-slide wedge method, where a small drop of blood was transferred from the sample tube via a stir stick and manually smeared. After drying, smears were stained with Fisher Healthcare PROTOCOL Hema 3 manual staining system and stat pack (Fisher Scientific; Ottawa, Ontario, Canada) according to the manufacturer's procedure and stored in slide boxes. Heterophils and lymphocytes were counted under an oil immersion lens (100 x magnification) until a total of 100 was reached. Heterophils were divided by lymphocytes to produce the H:L ratio as a measure of stress (Lentfer et al., 2015).

### Physiological biomarkers

At d 31, 1 bird/pen was randomly selected for collection of samples for biomarker analyses. Birds were weighed prior to euthanasia by cervical dislocation. One 5 g sample each of left *P.* major muscle and liver tissue was collected and snap frozen in liquid nitrogen prior to storage at −80⁰ C. Biomarkers assessed included TBA reactive substances, protein carbonyl, superoxide dismutase, glutathione peroxidase, glutathione, glutathione disulfide, catalase, and glutathione reductase.

#### TBA reactive substances (TBARS)

Approximately 50 and 25 mg of *P.* major muscle and liver tissue respectively were homogenized in 250 μL of radioimmunoprecipitation assay buffer, containing 1 μL of 1 M EDTA and centrifuged at 1600 x g at 4°C for 10 min. Supernatants were collected and analyzed using a TBARS kit (µM, Cayman Chemical Company, Ann Arbor, MI, item no. 10009055) and measured using microplate reader at 540 nm.

#### Protein carbonyl

Approximately 0.2 g of *P.* major muscle and liver tissue were homogenized in 1 mL of cold 50 mM potassium phosphate buffer, pH7, containing 1 mM EDTA and centrifuged at 10,000xg for 15 min at 4°C. Supernatants were removed and then incubated with 10 % streptomycin sulfate stock solution for 15 min at room temperature. They were then centrifuged at 6000 × g for 10 min at 4°C. Supernatants were analyzed using a kit (nmol/mL; Cayman Chemical Company, Ann Arbor, MI, item no. 10005020) and measured using a microplate reader at 360 nm.

#### Superoxide dismutase

Approximately 0.2 g of the *P.* major muscle and liver tissue were homogenized in 1 mL of cold 20 mM HEPES buffer, pH 7.2, containing 1 mM EGTA, 210 mM mannitol, and 70 mM sucrose, centrifuged at 1500 × g for 5 min at 4°C, analyzed using a super oxide dismutase kit (Units/mL; Cayman Chemical Company, Ann Arbor, MI, item no.706002) and measured using microplate reader at 440 nm.

#### Glutathione peroxidase

Approximately 0.2 g of the *P.* major muscle and liver tissue were homogenized in 1 mL of cold 50 mM Tris-HCL, pH 7.5, 5 mM EDTA, and 1 mM DTT, and the centrifuged at 10,000 × g for 15 min at 4 °C, the supernatant was analyzed using a kit (nmol/min/mL; Cayman Chemical Company, Ann Arbor, MI, item no. 703102) and measured using a microplate reader set at 340 nm.

#### Glutathione and glutathione disulfide

Approximately 0.2 g of the *P.* major muscle and liver tissue were homogenized in 1 mL of cold 50 mM phosphate buffer, pH 7, containing 1 mM EDTA. After centrifugation (10,000 × g for 15 min) at 4 °C, supernatants were analyzed using a kit (µM; Cayman Chemical Company, Ann Arbor, MI, item no. 703002) and measured using a microplate reader at 405 nm.

#### Catalase

Approximately 0.2 g of the *P.* major muscle and liver tissue were homogenized in 1 mL of cold 50 mM potassium phosphate buffer, pH 7, containing 1 mM EDTA. After centrifugation (10,000 × g for 15 min) at 4°C, supernatants were analyzed using a kit for catalase (nmol/min/mL; Cayman Chemical Company, Ann Arbor, MI, item no. 707002) and measured using a microplate reader at 540 nm.

#### Glutathione reductase

Approximately 0.2 g of the *P.* major muscle and liver tissue were homogenized in 1 mL of cold 50 mM potassium phosphate buffer, pH 7, containing 1 mM EDTA. After centrifugation (10,000 × g for 15 min) at 4°C, supernatants were analyzed using a kit for glutathione reductase (nmol/min/mL; Cayman Chemical Company, Ann Arbor, MI, item no. 703202) and measured using a microplate reader at 340 nm.

### Statistical analyses

Normality was tested using the UNIVARIATE procedure in SAS 9.4 (Cary, NC). Data were initially analyzed as a one-way ANOVA using the MIXED procedure and treatments ranked using Tukey-Kramer post Hoc test if different (*P* ≤ 0.05). Each room was an experimental block, with ten treatments per block. Each replicate pen was fed one of ten diets, for a total of five replicate pens for male broilers and four replicate pens for female broilers. As final BW (d 32) and BWG during the heat stress period (d 27 to 32) were influenced by BW at d 27, those results were analyzed as a one-way analysis of covariance (**ANCOVA**) with d 27 BW as the covariate. When *P* ≤ 0.05, data were analyzed as simplex lattice design in the ADX interface using SAS version 9.4 to generate a predictive model for the effects of Basal A (high S:L ratio), Basal B (high DigLys), and Basal C (high AME). All data were analyzed on a pen basis.

## Results

### Growth performance

The results for growth performance are displayed in Table 5, Table 6. Body weight gain over the finisher period (d 21 to 32) ranged between 0.703 and 0.929 kg for the males and 0.659 to 0.796 kg for the females (*P* < 0.05). The highest male BWG occurred in males fed the diet consisting of 33 % Basal B and 67 % Basal C. The one-way ANCOVA using d 27 BW as the covariate indicated no significant effect of diet on BWG in females, indicating the differences in female BWG during the finisher phase were due to the differences in growth during the adaptation period (d 21 to 27). Therefore, it was not possible to fit a model to optimize BWG of females thus male BWG data was used for optimization (Eqs. (1) and (2)). As d 27 BW affected BWG during the heat stress period, BWG for the whole finisher period was used for developing a recommendation, as to the authors’ knowledge, the model could not account for the dietary effects during the adaptation period on performance during heat stress.

**Table 5:** Effects of dietary starch:lipid (S:L) ratios, digestible amino acid content (DigLys), and AME on performance of male broilers during the finisher period with a period of heat stress (d 21 to 32) analyzed as a one-way ANOVA.^1^

	Treatments and respective mixing proportion of the basal diets											
	1	2	3	4	5	6	7	8	9	10	SEM	*P*-value
Basal A (high S:L)	0.00	0.00	0.00	0.00	0.33	0.33	0.33	0.67	0.67	1.00		
Basal B (high DigLys)	1.00	0.67	0.33	0.00	0.67	0.33	0.00	0.33	0.00	0.00		
Basal C (high AME)	0.00	0.33	0.67	1.00	0.00	0.33	0.67	0.00	0.33	0.00		

Body weight (kg)												
d 21	1.132	1.136	1.142	1.148	1.140	1.144	1.142	1.141	1.143	1.142	0.008	0.911
d 27	1.575^a^	1.590^a^	1.619^a^	1.601^a^	1.567^a^	1.568^a^	1.559^a^	1.458^b^	1.544^ab^	1.465^b^	0.021	<0.001
d 32^2^	2.002^ab^	2.036^a^	2.071^a^	2.023^ab^	1.988^ab^	1.999^a^	1.980^ab^	1.869^ab^	1.953^ab^	1.845^b^	0.022	0.009

BW gain (kg)												
d 21-27	0.443abc	0.454^ab^	0.476^a^	0.454^ab^	0.417^ab^	0.424^ab^	0.426^ab^	0.401^b^	0.317^c^	0.323^c^	0.013	<0.001
d 27-32^2^	0.425^ab^	0.448^a^	0.452^a^	0.421^ab^	0.422^ab^	0.431^a^	0.421^ab^	0.410^ab^	0.409^ab^	0.380^b^	0.011	0.001
d 21-32	0.868abc	0.900^ab^	0.929^a^	0.875abc	0.848abc	0.855abc	0.838^bc^	0.727^de^	0.711^de^	0.703^e^	0.018	<0.001

Feed intake (kg/bird)												
d 21-27	0.697^cd^	0.727bcd	0.784^ab^	0.809^a^	0.785^ab^	0.743abc	0.712bcd	0.7112bcd	0.661^cd^	0.735abcd	0.017	<0.001
d 27-32	0.809	0.834	0.870	0.871	0.894	0.863	0.884	0.841	0.836	0.838	0.019	0.092
d 21-32	1.506^b^	1.561^ab^	1.655^a^	1.681^a^	1.679^a^	1.606^ab^	1.596^ab^	1.552^ab^	1.497^b^	1.573^ab^	0.028	<0.001

F:G ratios												
d 21-27^3^	1.578^c^	1.586^c^	1.640^bc^	1.784^c^	1.672^bc^	1.748^bc^	1.865^b^	1.774^bc^	2.206^a^	2.290^a^	0.076	<0.001
d 27-32^3^	1.881^b^	1.900^b^	1.945^b^	2.067^ab^	2.078^ab^	2.015^ab^	2.088^ab^	2.028^ab^	2.051^ab^	2.198^a^	0.048	0.001
d 21-32^3^	1.741^e^	1.753^e^	1.806^de^	1.920^cd^	1.874cde	1.901^cd^	1.978^bc^	1.910^cd^	2.087^b^	2.243^a^	0.031	<0.001

Mortality %												
d 21-27	1.01	0.67	0.68	0.33	0.00	0.00	0.00	0.33	0.33	0.33	0.358	0.568
d 27-32	0.33	0.33	0.67	0.33	0.33	0.67	0.00	0.00	0.00	0.33	0.301	0.722
d 21-32	1.35	1.00	1.35	0.67	0.33	0.67	0.00	0.33	0.33	0.67	0.424	0.371

Abbreviations: F:G (feed-to-gain ratios).

1 Mean of replicates (*n* = 5) per treatment presented on a per bird basis.

2 Analysed as a one-way analysis of covariance (BW at d 27).

3 Mortality corrected.

abcd Different letters within rows indicate differences between treatments (*P* < 0.05).

**Table 6:** Effects of dietary starch:lipid (S:L) ratios, digestible amino acid content (DigLys), and AME on performance of female broilers during the finisher period with a period of heat stress (d 21 to 32) analyzed using one-way ANOVA.^1^

	Treatments and respective mixing proportion of the basal diets											
	1	2	3	4	5	6	7	8	9	10	SEM	*P*-value
Basal A (high S:L)	0.00	0.00	0.00	0.00	0.33	0.33	0.33	0.67	0.67	1.00		
Basal B (high DigLys)	1.00	0.67	0.33	0.00	0.67	0.33	0.00	0.33	0.00	0.00		
Basal C (high AME)	0.00	0.33	0.67	1.00	0.00	0.33	0.67	0.00	0.33	0.00		

Body weight (kg)												
d 21	1.041	1.047	1.044	1.037	1.048	1.046	1.032	1.038	1.041	1.036	0.011	0.988
d 27	1.453^ab^	1.486^a^	1.478^ab^	1.461^ab^	1.423^bc^	1.436^ab^	1.434^ab^	1.391^cd^	1.417^ab^	1.345^d^	0.016	<0.001
d 32^2^	1.807	1.825	1.840	1.833	1.794	1.793	1.874	1.727	1.751	1.695	0.024	0.150

BW gain (kg)												
d 21-27	0.412^ab^	0.439^a^	0.434^a^	0.425^ab^	0.375^bc^	0.390abc	0.374^bc^	0.355^bc^	0.354^bc^	0.309^c^	0.015	<0.001
d 27-32^2^	0.354	0.339	0.362	0.370	0.371	0.358	0.350	0.335	0.334	0.350	0.017	0.349
d 21-32	0.766^ab^	0.778^ab^	0.796^a^	0.796^a^	0.746abc	0.747abc	0.752^ab^	0.689^bc^	0.690^bc^	0.659^c^	0.019	0.002

F:G ratios												
d 21-27^3^	1.543^d^	1.548^d^	1.635^cd^	1.725^cd^	1.805^c^	1.715^c^	1.698^c^	1.723^c^	1.873^b^	2.178^a^	0.040	<0.001
d 27-32^3^	2.070^b^	2.071^b^	2.057^b^	2.082^b^	2.057^b^	2.143^b^	2.207^ab^	2.161^b^	2.199^ab^	2.352^a^	0.044	<0.001
d 21-32^3^	1.785^d^	1.790^d^	1.826^cd^	1.897^cd^	1.876^cd^	1.924bcd	1.956^bc^	2.064^ab^	1.945^bc^	2.181^a^	0.029	<0.001

Feed intake (kg/bird)												
d 21-27	0.636^d^	0.679abcd	0.709abc	0.731^a^	0.714^ab^	0.658bcd	0.635^d^	0.654^cd^	0.665bcd	0.666bcd	0.013	<0.001
d 27-32	0.740	0.745	0.748	0.772	0.749	0.767	0.763	0.735	0.763	0.768	0.014	0.563
d 21-32	1.376^b^	1.424^ab^	1.457^ab^	1.503^a^	1.464^ab^	1.425^ab^	1.398^ab^	1.389^b^	1.428^ab^	1.433^ab^	0.023	0.017

Mortality %												
d 21-27	0.00	0.00	0.37	0.37	0.00	0.74	0.00	0.00	0.00	0.00	0.214	0.206
d 27-32	0.00	0.37	0.00	0.00	0.00	1.10	0.74	0.00	0.00	0.37	0.408	0.530
d 21-32	0.00	0.37	0.37	0.37	0.00	1.84	0.74	0.00	0.00	0.37	0.398	0.075

Abbreviations: F:G (feed-to-gain ratios).

1 Mean of replicates (*n* = 5) per treatment presented on a per bird basis.

2 Analysed as a one-way analysis of covariance (BW at d 27).

3 Mortality corrected.

abcd Different letters indicate differences between treatments (*P* < 0.05).

**Fig. 1:**
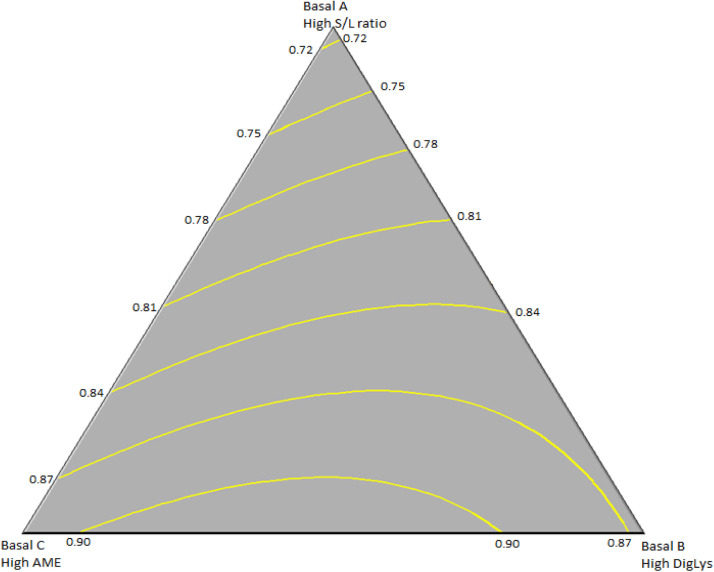
Surface model plot of the basal finisher diets on bodyweight gain (BWG; kg) of male broilers during the finisher period (d 21-32). R^2^=0.782.

Eq. (1): Male BWG (d 21 to 32; R^2^=0.782; Fig. 1):

BWG=0.708*Basal A + 0.862*Basal B + 0.889*Basal C + 0.175 * Basal A*Basal B + 0.173*Basal B*Basal C

Eq. (2): Male BWG during heat stress (d 27 to 32; R^2^=0.674)

BWG=0.391*Basal A + 0.429*Basal B + 0.428*Basal C + 0.097*Basal B*Basal C

Male and female F:G ratios were consistently lowest in birds fed a diet consisting of 100 % Basal B and with overall ratios of 1.741 and 1.785 respectively (Table 5, Table 6, *P* < 0.05). The modeled effects estimate lowest F:G ratios would occur in birds fed a diet composed of 100 % Basal B (Eqs. (3) and (4)).

**Fig. 2:**
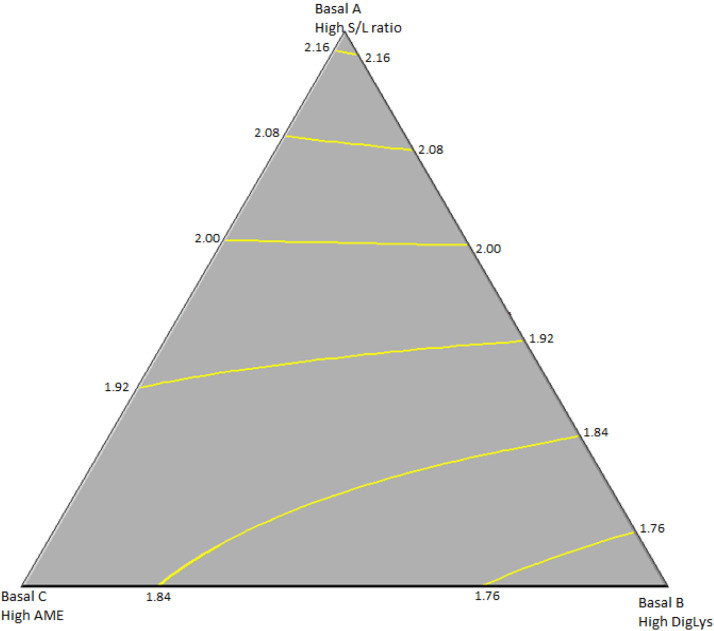
Surface model plot modeling the effects of basal finisher diets on feed-to-gain ratios of male broilers during the finisher phase (d 21-32). R^2^=0.739.

Eq. (3): Male F:G ratios (d 21 to 32; R^2^=0.739; Fig. 2):

F:G = 2.194*Basal A + 1.726*Basal B + 1.884*Basal C - 0.358*Basal A*Basal C

**Fig. 3:**
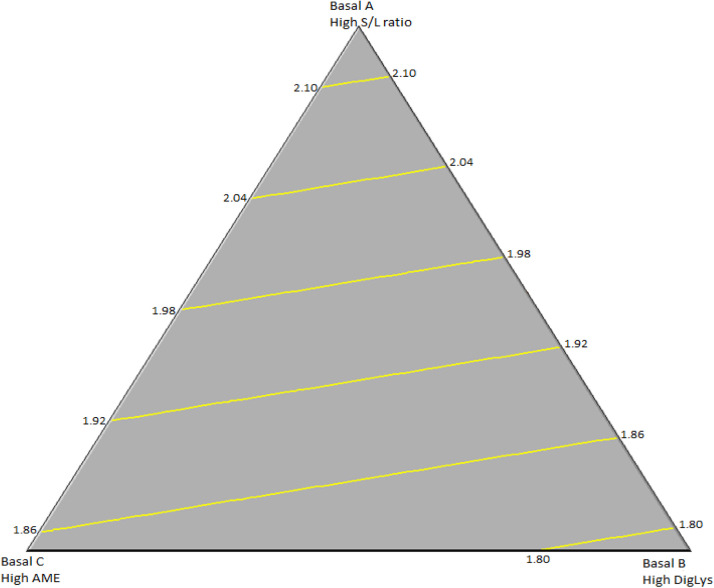
Surface model plot of the effects of the basal finisher diets on feed-to-gain ratios of female broilers during the finisher phase (d 21-32). R^2^=0.749.

Eq. (4): Female F:G ratios (d 21 to 32; R^2^=0.749; Fig. 3):

F:G = 2.139*Basal A + 1.783*Basal B + 1.850*Basal C

As inclusion of Basal A (high S:L ratio) resulted in lowest BWG and highest F:G ratios in both males and females, the optimum model considered the inclusion of that diet as zero. In the case of males, the combination of the diets to maximize BWG was predicted to occur in birds fed a diet comprised of 42.2 % Basal diet B and 57.8 % Basal diet C with a calculated BWG of 926 g from d 21 to 32 using a diet with a S:L ratio of 4:1, AME of 3,089 kcal/kg, and 1.01 % DigLys. Focusing on the heat stress period maximum male BWG of 453 g was estimated to occur in birds fed a diet composed of 50.5 % Basal B and 49.5 % Basal C for a diet with a S:L ratio of 4:1, AME of 3047 kcal/kg and 1.05 % DigLys.

### Carcass characteristics

Carcass yield results are shown for males and females in Table 7, Table 8, respectively. The diets affected the absolute live, carcass, and carcass cut-up weights in males and females (*P* < 0.05), with exception of *P.* minor in males and both *P.* minor and wings in females. In males, the diets affected yields (% live weight) of *P*. major and drumsticks (Eqs. (5) and (6)), while in females *P*. major, *P*. minor, and breast skin yield differed (*P* < 0.05; Eqs. (7)–(9)). The only parameter in males altered by the diets for yield as a percent of carcass weight was *P*. major while in females the *P*. major, back and rack and breast skin weights were affected (*P* < 0.05). The yields of male and female *P*. major, female *P*. minor, and male drums was highest in birds fed 100 % Basal B.

**Table 7:** Effects of dietary starch:lipid (S:L) ratios, digestible amino acid content (DigLys), and AME on carcass characteristics of heat stressed male broilers following a 5 day period of heat stress.^1^

	Treatments and respective mixing proportion of the basal diets											
	1	2	3	4	5	6	7	8	9	10	SEM	*P*-Value
Basal A (high S:L)	0.00	0.00	0.00	0.00	0.33	0.33	0.33	0.67	0.67	1.00		
Basal B (high DigLys)	1.00	0.67	0.33	0.00	0.67	0.33	0.00	0.33	0.00	0.00		
Basal C (high AME)	0.00	0.33	0.67	1.00	0.00	0.33	0.67	0.00	0.33	0.00		

Yield (g)												
Live weight	2,111abc	2031^bc^	2253^a^	2128abc	2112^ab^	2098abc	2175^ab^	2026^bc^	1962^c^	1992^c^	38.4	<0.001
Carcass	1495^ab^	1441^b^	1581^a^	1485^ab^	1497^ab^	1483	1513^ab^	1426^b^	1383^b^	1397^b^	27.8	<0.001
*Pectoralis* major	406.4^ab^	382.7^ab^	422.0^a^	374.9^ab^	405.2^ab^	393.7^ab^	395.8^ab^	375.3^b^	362.4^b^	358.0^b^	11.49	<0.001
*Pectoralis* minor	85.5	78.4	85.8	79.6	80.6	81.3	82.3	76.3	76.9	76.9	2.40	0.179
Breast skin	29.1^ab^	28.1^b^	34.7^a^	39.9^ab^	29.6^ab^	29.8^ab^	34.1^ab^	28.7^ab^	28.3^ab^	29.2^ab^	1.45	0.003
Thighs	251.8^ab^	236.5^b^	264.2^a^	253.8^ab^	253.1^ab^	246.9^ab^	256.2^ab^	242.8^ab^	231.2^b^	236.8^b^	5.34	<0.001
Drums	211.6^ab^	205.4abc	214.4^a^	207.4abc	206.9abc	204.8abc	205.6abc	196.1^abc^	189.7^c^	194.6^bc^	4.07	<0.001
Wings	163.6^ab^	155.9^b^	169.0^a^	162.7^ab^	162.2^ab^	162.9^ab^	164.4^ab^	157.6^ab^	153.1^b^	154.9^b^	2.75	<0.001
Back and rack	347.7^ab^	346.5^ab^	383.2^a^	361.5^ab^	356.7^ab^	358.7^ab^	370.2^ab^	340.7^b^	335.4^b^	343.2^b^	8.15	0.001

Yield (% live weight)												
Carcass	70.80	70.91	70.21	69.84	71.03	70.67	69.52	70.46	70.49	70.18	0.456	0.367
*Pectoralis* major	19.25^a^	18.77^ab^	18.73^ab^	17.63^b^	19.19^a^	18.75^ab^	18.19^ab^	18.62^ab^	18.39^ab^	17.97^ab^	0.316	0.007
*Pectoralis* minor	3.92	3.87	3.81	3.75	3.88	3.88	3.78	3.78	3.92	3.87	0.095	0.928
Breast skin	1.37	1.39	1.54	1.55	1.41	1.43	1.58	1.41	1.44	1.47	0.065	0.243
Thighs	11.93	11.64	11.74	11.93	12.00	11.77	11.79	12.02	11.79	11.88	0.151	0.755
Drums	10.04^ab^	10.14^a^	9.52^ab^	9.76^ab^	9.81^ab^	9.77^ab^	9.45^b^	9.69^ab^	9.70^ab^	9.80^ab^	0.136	0.029
Wings	7.75	7.71	7.51	7.66	7.69	7.77	7.57	7.80	7.84	7.80	0.093	0.392
Back and rack	16.47	17.06	17.02	17.01	16.88	17.08	16.98	16.84	17.14	17.23	0.248	0.701

Yield (% carcass weight)												
*Pectoralis* major	27.24^a^	26.47^ab^	26.68^ab^	25.25^b^	27.10^ab^	26.54^ab^	26.16^ab^	26.43^ab^	26.07^ab^	25.61^ab^	0.385	0.010
*Pectoralis* minor	5.52	5.45	5.41	5.38	5.36	5.51	5.45	5.36	5.57	5.48	0.132	0.978
Breast skin	1.95	1.94	2.20	2.22	1.96	2.02	2.26	1.99	2.03	2.08	0.083	0.328
Thighs	16.84	16.42	16.72	17.06	16.88	16.65	16.94	17.05	16.72	16.92	0.200	0.474
Drums	14.16	14.29	13.57	13.97	13.79	13.82	13.58	13.76	13.76	13.95	0.176	0.128
Wings	10.98	10.87	10.70	10.96	10.81	10.99	10.88	11.06	11.10	11.11	0.131	0.432
Back and rack	23.27	24.05	24.45	24.32	23.72	24.14	24.45	23.87	24.31	24.56	0.304	0.118

1 Mean of replicates (*n* = 5) per treatment presented on a per bird basis.

abc Different letters indicate differences between treatments (*P* < 0.05).

**Table 8:** Effects of dietary starch:lipid (S:L) ratios, digestible amino acid content (DigLys), and AME on carcass characteristics of heat stressed female broilers following a 5 day period of heat stress.^1^

	Treatments and respective mixing proportion of the basal diets											
	1	2	3	4	5	6	7	8	9	10	SEM	*P*-Value
Basal A (high S:L)	0.00	0.00	0.00	0.00	0.33	0.33	0.33	0.67	0.67	1.00		
Basal B (high DigLys)	1.00	0.67	0.33	0.00	0.67	0.33	0.00	0.33	0.00	0.00		
Basal C (high AME)	0.00	0.33	0.67	1.00	0.00	0.33	0.67	0.00	0.33	0.00		

Yield (g)												
Live weight	1895^a^	1909^a^	1902^a^	1898^a^	1860^ab^	1857^ab^	1848^ab^	1857^ab^	1829^ab^	1754^b^	26.4	0.002
Carcass	1360^a^	1353^a^	1336^a^	1334^ab^	1322^ab^	1314^ab^	1318^ab^	1311^ab^	1289^ab^	1233^b^	20.7	0.014
*Pectoralis* major	369.0^a^	358.1^ab^	339.3^ab^	330.3^ab^	351.2^ab^	340.6^ab^	336.5^ab^	341.9^ab^	335.3^ab^	321.8^b^	7.76	0.002
*Pectoralis* minor	80.3	80.0	76.2	72.4	77.6	73.9	74.2	73.8	76.8	72.3	2.03	0.059
Breast skin	27.0^b^	28.9^ab^	29.5^ab^	33.9^a^	28.9^ab^	31.4^ab^	31.7^ab^	29.4^ab^	27.0^b^	26.3^b^	1.29	0.001
Thighs	232.3^a^	229.7^a^	228.8^ab^	230.6^a^	225.3^ab^	223.4^ab^	226.9^ab^	221.9^ab^	220.4^ab^	208.9^b^	4.38	0.014
Drums	178.5^a^	180.5^a^	178.4^ab^	177.4^ab^	173.9^ab^	174.8^ab^	171.2^ab^	177.8^ab^	172.2^ab^	164.8^b^	3.15	0.020
Wings	147.7	146.9	145.4	146.1	143.7	144.8	145.4	144.3	142.3	138.2	2.23	0.067
Back and rack	318.1^ab^	320.9^ab^	330.3^a^	337.1^a^	312.2^ab^	318.6^ab^	325.6^ab^	319.7^ab^	308.2^ab^	295.4^b^	6.71	0.003

Yield (% live weight)												
Carcass	71.79	70.91	70.23	70.29	71.07	70.79	71.33	70.65	70.57	70.79	0.657	0.865
*Pectoralis* major	19.45^a^	18.74abc	17.84^bc^	17.40^c^	18.87abc	18.34abc	18.17abc	18.40abc	18.34^ab^	18.50abc	0.330	0.003
*Pectoralis* minor	4.25^a^	4.19^ab^	4.01^ab^	3.82^b^	4.18^ab^	3.97^ab^	4.02^ab^	3.98^ab^	4.20^ab^	4.15^ab^	0.101	0.026
Breast skin	1.42^c^	1.52^bc^	1.55^bc^	1.79^a^	1.55abc	1.68abc	1.73^ab^	1.58abc	1.47^bc^	1.51^bc^	0.065	0.001
Thighs	12.28	12.04	12.04	12.14	12.10	12.04	12.27	11.95	12.06	11.97	0.186	0.952
Drums	9.44	9.47	9.39	9.36	9.37	9.44	9.36	9.57	9.41	9.45	0.135	0.988
Wings	7.82	7.70	7.65	7.69	7.75	7.80	7.89	7.77	7.80	7.93	0.107	0.754
Back and rack	16.78	16.83	17.34	17.73	16.75	17.16	17.61	17.21	16.87	16.94	0.267	0.097

Yield (% carcass weight)												
*Pectoralis* major	27.11^a^	26.42^ab^	25.39^ab^	24.76^b^	26.51^ab^	25.90^ab^	24.45^ab^	26.09^ab^	25.95^ab^	26.09^ab^	0.370	0.002
*Pectoralis* minor	5.90	5.92	5.71	5.43	5.82	5.62	5.65	5.62	5.96	5.85	0.127	0.099
Breast skin	1.99^c^	2.13^bc^	2.20abc	2.54^a^	2.21abc	2.38abc	2.42^ab^	2.24abc	2.07^bc^	2.12^bc^	0.090	<0.001
Thighs	17.11	16.98	17.14	17.28	17.07	17.00	17.21	16.93	17.11	16.92	0.209	0.968
Drums	13.16	13.35	13.36	13.31	13.18	13.30	13.13	13.56	13.37	13.37	0.177	0.869
Wings	10.89	10.86	10.90	10.95	10.84	11.03	11.06	10.99	11.06	11.21	0.136	0.730
Back and rack	23.34^b^	23.72^ab^	24.69^ab^	25.22^a^	23.64^b^	24.25^ab^	24.70^ab^	24.35^ab^	23.92^ab^	23.96^ab^	0.313	0.008

1 Mean of replicates (*n* = 4) per treatment presented on a per bird basis.

abcd Different letters indicate differences between treatments (*P* < 0.05).

Eq. (5): Male *P*. major yield (% live weight; R^2^=0.20)

*P*. major = 17.9*Basal A + 19.1*Basal B + 17.7*Basal C + 3.9*Basal A*Basal C

Eq. (6): Male drumstick yield (% live weight; R^2^=0.16)

Drumsticks = 9.8*Basal A + 10.0*Basal B + 9.7*Basal C – 1.5*Basal A*Basal B

Eq. (7): Female *P*. major yield (% live weight; R^2^=0.41)

*P*. major = 18.4*Basal A + 19.3*Basal B + 17.5*Basal C – 2.8*Basal A*Basal B +3.0*Basal A*Basal C

Eq. (8): Female *P*. minor yield (% live weight; R^2^=0.40)

*P*. minor = 4.3*Basal A + 4.3*Basal B + 3.9*Basal C – 1.2*Basal A*Basal B

Eq. (9): Female breast skin yield (% live weight; R^2^=0.44)

Breast skin = 1.4*Basal A + 1.4*Basal B + 1.7*Basal C + 1.0*Basal A*Basal B

Maximum yield of male and female *P*. major, female *P*. minor, and male drumsticks were calculated to occur in birds fed 100 % Basal B. Lowest yield of the *P*. majors, male drumsticks, and female *P*. minor were estimated to occur in birds fed a diet consisting of 100 % Basal C. Breast skin yields of females was estimated to be highest in females fed 100 % Basal C.

### Nutrient retention

Treatment affected DM and N retention in male and female broilers (*P* < 0.05). The N corrected AME of the diets ranged between 2696 (100 % Basal A) and 3419 (100 % Basal B) kcal/kg in the males and between 2683 (100 % Basal A) and 3434 (100 % Basal B) kcal/kg in the females. DM retention (Eqs. (10) and (11)) and N retention (Eqs. (12) and (13)) were affected by the diets in both males and females (*P* < 0.05). The results for nutrient retention are displayed in Table 9, Table 10.

**Table 9:** Effects of dietary starch:lipid (S:L) ratios, digestible amino acid content (DigLys), and AME on nutrient retention of male broilers during heat stress analyzed using one-way ANOVA.^1^

	Treatments and respective mixing proportion of the basal diets												
	1	2	3	4	5	6	7	8	9	10	SEM	*P*-Value	
Basal A (high S:L)	0.00	0.00	0.00	0.00	0.33	0.33	0.33	0.67	0.67	1.00			
Basal B (high DigLys)	1.00	0.67	0.33	0.00	0.67	0.33	0.00	0.33	0.00	0.00			
Basal C (high AME)	0.00	0.33	0.67	1.00	0.00	0.33	0.67	0.00	0.33	0.00			

Dry matter (%)	65.60^d^	71.46^bc^	72.51^b^	77.35^a^	68.31^cd^	71.50^bc^	78.65^a^	70.64^bc^	73.76^b^	68.65^cd^	0.626		<0.001
Nitrogen (%)	69.16^b^	71.33^ab^	68.30^b^	68.50^b^	75.66^a^	71.80^ab^	75.77^a^	72.22^ab^	67.38^b^	66.81^b^	1.008		<0.001
AME (kcal/kg)	3,094^ef^	3,282^cd^	3,338^bc^	3,511^a^	3,082^ef^	3,219^d^	3,446^ab^	3,002^f^	3,182^de^	2,787^g^	19.8		<0.001
AMEn (kcal/kg)	2,964^d^	3,165^bc^	3,235^b^	3,419^a^	2,946^d^	3,124^c^	3,342^a^	2,890^d^	3,080^c^	2,696^e^	18.7		<0.001

1 Mean of replicates (*n* = 2) per treatment presented on a per bird basis.

abcdefg Different letters indicate differences between treatments (*P* < 0.05).

**Table 10:** Effects of dietary starch:lipid (S:L) ratios, digestible amino acid content (DigLys), and AME on nutrient retention of female broilers during heat stress analyzed using one-way ANOVA.^1^

	Treatments and respective mixing proportion of the basal diets											
	1	2	3	4	5	6	7	8	9	10	SEM	*P*-Value
Basal A (high S:L)	0.00	0.00	0.00	0.00	0.33	0.33	0.33	0.67	0.67	1.00		
Basal B (high DigLys)	1.00	0.67	0.33	0.00	0.67	0.33	0.00	0.33	0.00	0.00		
Basal C (high AME)	0.00	0.33	0.67	1.00	0.00	0.33	0.67	0.00	0.33	0.00		

Dry matter (%)	65.95^e^	68.99^d^	70.43^cd^	77.63^a^	64.63^e^	72.08^bc^	76.94^a^	71.80^bc^	73.36^b^	69.18^d^	0.355	<0.001
Nitrogen (%)	61.12^c^	67.54abc	64.49^bc^	68.50^ab^	64.17^bc^	67.68^abc^	72.38^a^	69.06^ab^	64.67^bc^	63.55^bc^	1.272	<0.001
AME (kcal/kg)	3,073^d^	3,238^c^	3,281^c^	3,526^a^	2,914^f^	3,215^c^	3,368^b^	2,998^e^	3,137^d^	2,770^g^	13.5	<0.001
AMEn (kcal/kg)	2,959^c^	3,127^bc^	3,194^c^	3,434^a^	2,799^d^	3,116^bc^	3,269^b^	2,882^cd^	3,039^bc^	2,683^e^	12.6	<0.001

1 Mean of replicates (*n* = 2) per treatment presented on a per bird basis.

abcdefg Different letters indicate differences between treatments (*P* < 0.05).

Eq. (10): DM retention in males (%; R^2^=0.74)

DM retention= 68.8*Basal A + 67.0*Basal B + 76.4*Basal C + 15.6*Basal A*Basal B + 35.7*Basal A*Basal C

Eq. (11): DM retention in females (%; R^2^=0.68)

DM retention= 69.8*Basal A + 66.1*Basal B + 77.0*Basal C + 18.7*Basal A*Basal B + 32.5*Basal A*Basal C

Eq. (12): N retention in males (%; R^2^=0.46)

N retention= 67.1*Basal A + 68.3*Basal B + 72.1*Basal C + 27.5*Basal A*Basal C

Eq. (13): N retention in females (%; R^2^=0.26)

N retention= 64.0*Basal A + 63.5*Basal B + 67.8*Basal C + 21.7*Basal A*Basal B

Maximum N retention in males was predicted to occur in males fed a combination of 40.9 % Basal A and 59.1 % Basal C with a S:L ratio of 6.2:1, AME of 3,096 kcal/kg, and 0.80 DigLys. In females, maximum N retention was estimated to occur when fed a mixture of 51.2 % Basal A and 48.8 % Basal B with an S:L ratio of 8:1, AME of 2,800 kcal/kg, and 1.04 % DigLys.

### Biomarkers

Results for analyses of biomarkers and oxidative stress parameters are shown in Table 11. Glutathione reductase activity was affected by the diets in the livers of male broilers with the highest levels found in birds fed 100 % Basal C and lowest in bird fed 67 % Basal B and 33 % Basal C (*P* < 0.05; Eq. (14)). The diets affected glutathione peroxidase activity in the livers of male and female birds (*P* < 0.05; Eqs. (15) and (16)). Highest glutathione peroxidase activity was detected in livers of males fed 100 % Basal C and females fed 67 % Basal A and 33 % Basal B. Lowest levels were detected in males fed 67 % Basal A and 33 % Basal C and females fed 33 % Basal A, 33 % Basal B, and 33 % Basal C. Glutathione concentrations in the male breast and female livers were altered by the diets with the highest levels in the males fed the diets consisting of 100 % Basal C and females fed 67 % Basal A and 33 % Basal C (*P* < 0.05 Eqs. (17) and (18)). The diets affected catalase activity in the livers of male and female birds with the highest activity in males fed 100 % Basal A and females fed 33 % Basal A and 67 % Basal C (*P* < 0.05 Eqs. (19) and (20)). Superoxide dismutase activity and protein carbonyl concentrations were not altered by the diets (*P* > 0.05). The concentrations of TBARS were altered in male breast tissue with highest concentrations in males fed 33 % Basal A, 33 % Basal B, and 33 % Basal C (*P* < 0.05 Eq. (21)).

**Table 11:** Effects of dietary starch:lipid (S:L) ratios, digestible amino acid content (DigLys), and AME on oxidative damage and biomarker activity in male and female broilers analyzed using one-way ANOVA.^1^

	Treatments and respective mixing proportion of the basal diets											
	1	2	3	4	5	6	7	8	9	10	SEM	*P*-Value
Basal A (high S:L)	0.00	0.00	0.00	0.00	0.33	0.33	0.33	0.67	0.67	1.00		
Basal B (high DigLys)	1.00	0.67	0.33	0.00	0.67	0.33	0.00	0.33	0.00	0.00		
Basal C (high AME)	0.00	0.33	0.67	1.00	0.00	0.33	0.67	0.00	0.33	0.00		

Glutathione reductase (nmol/min/mL)												
Male breast	12.14	9.76	12.39	11.63	8.83	6.19	9.34	6.37	6.96	8.74	2.711	0.690
Female breast	7.64	6.02	8.06	9.33	7.64	8.23	7.21	9.84	13.15	6.53	2.390	0.681
Male liver	74.60^bc^	61.36^c^	89.05^abc^	85.33abc	117.12^ab^	104.22^abc^	107.56^abc^	82.960^abc^	102.22^abc^	125.72^a^	10.555	0.003
Female liver	104.42	105.78	105.19	98.48	93.75	102.39	83.78	99.14	89.98	88.22	8.180	0.528

Glutathione peroxidase (nmol/min/mL)												
Male breast	11.4	9.3	13.3	14.9	9.3	12.0	12.0	8.7	8.4	7.7	3.61	0.911
Female breast	17.4	7.1	8.7	13.8	9.8	7.0	9.9	6.5	12.3	6.5	2.85	0.148
Male liver	216.1^bcd^	207.6^cd^	273.8^a^	277.4^a^	240.8^abc^	265.6^a^	249.3^abc^	241.5^abc^	188.4^d^	259.6^ab^	9.39	<0.001
Female liver	262.6^a^	246.1^a^	249.0^a^	241.1^a^	228.2^ab^	178.0^b^	226.0^ab^	266.7^a^	235.8^a^	252.2^a^	11.41	<0.001

Catalase (nmol/min/mL)												
Male breast	2394	2299	2309	3022	1863	2731	2951	2519	2618	2914	373.3	0.507
Female breast	2387	2214	2509	2334	1912	3047	2798	2673	2680	2549	381.8	0.704
Male liver	14,167^bc^	11,252^d^	14,985^abc^	14,703^abc^	13,430^c^	15,958^a^	16,104^a^	15,436^ab^	13,758^bc^	14,836^abc^	361.0	<0.001
Female liver	13,975^b^	15,167^ab^	13,946^b^	15,050^ab^	14,678^ab^	15,398^ab^	14,738^ab^	14,602^ab^	15,538^a^	15,711^a^	319.5	0.002

Glutathione (µM)												
Male breast	256.0^ab^	236.6^b^	233.7^b^	273.5^a^	249.7^ab^	247.1^ab^	238.5^b^	239.2^b^	266.4^ab^	267.3^ab^	7.23	0.001
Female breast	257.3	241.2	252.3	230.8	249.6	244.3	234.6	245.4	234.4	239.1	6.04	0.074
Male liver	493.1	474.2	481.7	458.0	498.7	488.5	495.3	465.9	497.8	493.0	15.28	0.562
Female liver	469.6	463.6	501.2	496.3	494.4	479.7	474.2^a^	512.5	446.42	475.1	13.82	0.059

Superoxide dismutase (U/mL)												
Male breast	1.76	1.93	1.95	1.89	1.94	1.83	1.83	1.76	1.85	1.86	0.104	0.907
Female breast	1.88	1.77	1.86	1.95	1.97	1.85	1.88	1.86	1.87	1.80	0.103	0.957
Male liver	0.64	0.73	0.67	0.66	0.76	0.62	0.46	0.55	0.55	0.59	0.067	0.118
Female liver	0.49	0.48	0.63	0.46	0.73	0.43	0.60	0.46	0.52	0.61	0.086	0.298

Protein carbonyl (nmol/mg)												
Male breast	3.68	11.91	5.30	8.57	10.50	7.52	9.18	6.95	8.57	9.32	1.933	0.162
Female breast	6.84	12.64	6.89	9.25	10.18	8.23	10.48	10.80	10.50	14.32	2.500	0.542
Male liver	13.23	9.87	11.32	15.56	9.45	8.63	15.45	8.75	16.27	11.80	4.667	0.958
Female liver	18.73	10.36	13.20	10.02	11.50	9.22	11.95	8.25	5.89	11.30	3.593	0.538

TBARS/lipid peroxidation (µM)												
Male breast	3.36^ab^	1.09^b^	1.18^b^	1.27^ab^	2.45^ab^	5.45^a^	1.82^ab^	5.00^ab^	1.67^ab^	1.82^ab^	0.891	0.006
Female breast	16.00	3.55	3.73	4.63	9.36	2.91	4.09	3.45	8.64	5.73	4.396	0.562
Male liver	7.36	3.91	8.73	9.00	10.18	8.09	10.91	9.09	8.36	5.91	2.266	0.611
Female liver	7.82	9.27	7.64	7.00	9.45	9.82	7.91	6.45	7.91	6.09	2.047	0.939

Abbreviations: TBARS (Thiobarbituric acid reactive substances).

1 Mean of replicates (male: *n* = 5, female *n* = 4) per treatment presented on a per pen basis.

abcd Different letters indicate differences between treatments (*P* < 0.05).

Eq. (14): Glutathione reductase in the male liver (nmol/min/mL; R^2^=0.22)

GRD= 110.0*Basal A + 66.2*Basal B + 88.6*Basal C + 139.8*Basal A*Basal C

Eq. (15): Glutathione peroxidase activity in male livers (nmol/min/mL; R^2^=0.25)

GPX= 218.2*Basal A + 253.4*Basal B + 231.0*Basal C + 141.4*Basal A*Basal B

Eq. (16): Glutathione peroxidase activity in female livers (nmol/min/mL; R^2^=0.29)

GPX= 252.3*Basal A + 229.1*Basal B + 260.2*Basal C – 155.2*Basal B*Basal C

Eq. (17): Glutathione concentrations in male livers (µM; R^2^=0.04)

GSG= 479.5*Basal A + 487.1*Basal B + 487.2*Basal C

Eq. (18): Glutathione concentrations in female livers (µM; R^2^=0.13)

GSG= 482.1*Basal A + 490.6*Basal B + 491.6*Basal C – 123.1*Basal A*Basal C

Eq. (19): Catalase activity in male livers (nmol/min/mL; R^2^=0.55)

CAT= 12,159*Basal A + 13,559*Basal B + 15,417*Basal C + 7,756*Basal A*Basal B + 5,765*Basal B*Basal C

Eq. (20): Catalase activity in female livers (nmol/min/mL; R^2^=0.15)

CAT= 15,395*Basal A + 15,037*Basal B + 14,874*Basal C – 4,001*Basal A*Basal B

Eq. (21): Thiobarbituric acid reactive substances concentrations in male livers (µM; R^2^=0.19)

TBARS= 0.60*Basal A + 2.92*Basal B + 4.01*Basal C

### H:L ratios

No effects due to the diets were detected in the H:L ratios of male or female birds (*P* > 0.05). The results of these measures are summarized in Table 12.

**Table 12:** Effects of dietary starch:lipid (S:L) ratios, digestible amino acid content (DigLys), and AME on H:L ratios of broilers during heat stress analyzed using one-way ANOVA.^1^

	Treatments and respective mixing proportion of the basal diets											
	1	2	3	4	5	6	7	8	9	10	SEM	P-Value
Basal A (high S:L)	0.00	0.00	0.00	0.00	0.33	0.33	0.33	0.67	0.67	1.00		
Basal B (high DigLys)	1.00	0.67	0.33	0.00	0.67	0.33	0.00	0.33	0.00	0.00		
Basal C (high AME)	0.00	0.33	0.67	1.00	0.00	0.33	0.67	0.00	0.33	0.00		

Male H:L	0.826	0.876	0.888	0.832	0.862	0.900	0.907	0.861	0.878	0.847	0.0453	0.939
Female H:L	0.901	0.924	0.865	0.864	0.911	0.931	0.946	0.869	0.909	0.892	0.0295	0.494

Abbreviations: H:L (Heterophil to Lymphocyte ratio).

1 Mean of replicates (*n* = 5 for male; *n* = 4 for female).

## Discussion

There was clear evidence that increasing inclusion of Basal A (high S:L ratio) resulted in poorer performance outcomes in the broilers, with both reduced BWG and increased F:G ratios. This was likely due to a combination of the high S:L ratio (Khoddami et al., 2018), the reduced fat/energy content (Rand et al., 1958), and low amino acid content of the diet. There was a synergistic effect between Basal diets A and B (high DigLys), and B and C (high AME) as both combinations resulted in improved male BWG. In the case of the interaction between Basals A and B, it negatively affected BWG most likely due to the combination of increased CP content and S:L of the two diets increasing heat increment (Soares et al., 2020). The high proportion of energy coming from dietary fat content may have also improved performance as supplementing fat into diets has been shown to reduce dietary rate of passage which can enhance digestion and utilization of dietary energy and other nutrients (Mateos et al., 1982). Fat also has a lower heat increment compared to protein or carbohydrates (Fuller and Rendon, 1977). Basal B had an increased dietary electrolyte balance of 255 milliequivalent/kg (mEq/kg) compared to 222 mEq/kg in the other two basal diets which may have aided in improving performance of the broilers during the heat stress period (Popoola et al., 2020). As differences in female BWG were due to the diets affecting growth during the adaptation phase (d 21 to 27) with no observed dietary effects during heat stress the effect of the diets on female BWG were not modeled.

During heat stress, male BWG may be further improved by reducing the CP content of the diet and supplementing with higher inclusions of crystalline amino acids as CP has a higher heat increment than either fat or carbohydrates (Musharaf and Latshaw, 1999) which could reduce feed intake due to increased heat generation. Attia et al. (2020) observed that reducing CP content of diets from 18 to 15 % with supplementation of methionine and lysine did not affect growth performance and meat yield while also improving N retention of heat stressed broilers. Chrystal et al. (2020) observed that reducing CP content of diets from 21.0 % to 16.5 % improved AMEn and amino acid digestibility by 9.89 % and 9.14 % respectively although no effects were observed for BWG. The broilers fed a diet consisting of 100 % Basal B with the highest amino acid density (1.30 % DigLys) had the lowest F:G ratios regardless of potential effects of high CP of the diet (Musharaf and Latshaw, 1999). This suggests that increasing amino acid density is more crucial than increasing energy or S:L ratio in improving F:G under heat stress.

Based on the observed effects on carcass characteristics, while increasing inclusion of Basal C improved BWG, it also reduced male and female breast yield as a percent of live weight. This is likely due to the high energy content of the diet relative to amino acid content resulting in reduced muscle and increased fat deposition (Johnson et al., 2020). The diet composed of 100 % Basal B (highest DigLys density and lowest AMEn) also had the highest percent breast meat yield in both males and females which may indicate that energy intake does not limit muscle deposition with high amino acid intake within the limits of this experiment (Eits et al., 2002). There was also a negative interaction between Basals A and B which affected yields of female *P*. major and *P*. minor, along with male drumsticks (% live weight) which could be related to the high nitrogen content of Basal B increasing energy costs of excretion (Maharjan et al., 2021) combined with the high proportion of energy coming from starch in Basal A increasing heat increment of the diet (Fuller and Rendon, 1977). Those two factors may have interacted and increased energy utilization which could have otherwise been used to growth. There was a positive interaction between Basals A and C increasing percent of *P*. major yield in males and females. This could have been due to the combination of the energy content of Basal C and the carbohydrate content of Basal A having a protein sparing effect, allowing the amino acids to be used for muscle deposition which could then be used to deposit muscle (Fuller and Crofts, 1977; Kyriazikis and Emmans, 1992). However, the R^2^ of the dietary effect was low (<0.50), and thus, there does not appear to be a strong relation between the diets and meat yield. The highest estimated yield for both sexes was predicted to occur when birds were fed a diet composed of 100 % Basal B which may mean further improvements to meat yield may be possible by feeding diets with higher amino acid concentrations. Females had a stronger response compared to the males for meat yield. Males showed a difference of 1.2 % from lowest to highest estimated meat yield whereas females had a difference of 1.9 % which might be indicative that females responded more to increased amino acid content of the diets leading to increased muscle deposition.

Increasing inclusion of Basal C linearly reduced breast meat yield (as a percent of live weight) relative to Basal B in males and females perhaps due to the lower amino acid content of the diet leading to increased fat deposition. As Basal B had the largest improvement to F:G ratios and increasing inclusion for BWG during heat stress, it may be desirable to formulate diets closer in composition to Basal B for more efficient growth and higher meat yield at the cost of lower body weights at slaughter. However, most cost-effective diet formulation, may vary based on the cost of feed compared to the value of the product as a system where producers are paid based on slaughter weights could benefit more from the increased BW, whereas a vertically integrated system may desire more meat yield and efficiency. Achieving the composition of Basal B may be challenging in practical feed formulation because of its low energy content and high digestible amino acid content.

As Basal B had the highest content of filler used to meet energy specifications (sand and silica dioxide) at 6.5 % followed by Basal A at 4.8 %, this is likely part of the reason that DM retention was lower in those diets compared to Basal C which contained 1.2 %. There was also an interaction between Basals A and B, and Basals A and C which increased the percent dry matter retention and may be indicative of the reduced S:L ratio associated with Basals B and C improving overall diet digestibility in the heat stressed birds (Khoddami et al., 2018). There was an interaction between Basals A and C increasing N retention. The higher fat content of Basals B and C may have slowed dietary rate of passage in the gut improving nutrient digestibility and retention (Mateos et al., 1982). This is likely why Basal C had a higher base CP retention compared to the other two diets due to it having the highest dietary fat content. The AMEn of Basals B and C were higher than the formulated values while Basal A was lower. This may have been due to the effects of heat stress altering gastrointestinal activity combined with the differences in nutrient content such as dietary fat improving digestibility of other nutrients (Mateos et al., 1982), and free amino acid content (Morales et al., 2020).

Overall, results for biomarker measures were quite variable and except for catalase activity in the liver of male broilers, the generated models had a R^2^<0.50. Basal B interacted with Basals A, and C increasing catalase activity in the liver of broilers. This is likely due to the amino acid content of the diet, specifically methionine which is converted to S-adenosylmethionine which acts as a methyl donor, possibly influencing catalase activity (Lugata et al., 2022). Glutathione reductase had the highest activity in the livers of males fed a diet of 100 % Basal C which may be indicative of high glutathione activity, possibly due in part to the high starch content of the diet triggering an inflammation response (Zhang et al., 2022). Glutathione peroxidase was highest in the livers of male birds fed 100 % Basal C and lowest in those fed 67 % Basal A and 33 % Basal C, while in females it was lowest in birds fed 33 % Basals A, B, and C. However the modeled responses fit was poor (R^2^<0.50). Modeled effects of the diets on glutathione fit the diets poorly for the male breast (R^2^=0.04) and female liver (R^2^=0.13), along with TBARS in male breast (R^2^=0.19) which is likely due to high variability of the results reducing accuracy.

The H:L ratios of male and female broilers were not affected by the diets under heat stress conditions. Apparently, while the diets affected broiler performance, they did not alter the stress response of birds exposed to heat stress.

Improvements to this model could entail reducing the duration of the adaptation period, which could limit the effects of the diets during that period. Particularly as male and female BWG during the finisher period was impacted by the effects of the diets during the adaptation period, reducing the duration may result in better modeling and more accurately optimizing broiler performance. However, doing so may result in the animals rejecting feed which could increase variation such as for Basal A due to the high inclusion of corn starch (22.04 %).

In conclusion, growth performance of heat stressed broilers was influenced by feeding diets with differing AME, amino acid density, and S:L ratios. Diets with high AME and amino acid content generally resulted in improved performance relative to diets with high S:L ratio. H:L ratios were not affected by diet, and the biomarker results suggest that diet composition may not be a reliable predictor of oxidative stress in heat-stressed broilers. Based on the modeled effects, male BWG during the finisher phase and under cyclical heat stress conditions is maximized when fed, a diet with an AME of 3,089 kcal/kg, 1.01 % DigLys, and a S:L ratio of 4:1 while female performance was not affected. Although an optimal recommendation for male and female F:G ratio could not be determined, results suggest that diet with high amino acid density in the environmental conditions utilized in this experiment may be beneficial.

## Disclosures

J. C. de Paula Dorigam and R. Whelan are/were employed by Evonik Operation GmbH (Hanau-Wolfgang, Essen, Germany). No other authors have conflicts of interest.

## Declaration of competing interest

Karen Schwean-Lardner reports financial support was provided by Evonik Operations GmbH. Karen Schwean-Lardner reports financial support was provided by Natural Sciences and Engineering Research Council of Canada. Juliano C. de Paula Dorigam reports a relationship with Evonik Operations GmbH that includes: employment. Rose Whelan reports a relationship with Evonik Operations GmbH that includes: employment. If there are other authors, they declare that they have no known competing financial interests or personal relationships that could have appeared to influence the work reported in this paper.

## References

[bib0001] AOAC (2016).

[bib0002] Attia Y.A., Hassan S.S. (2017). Broiler tolerance to heat stress at various dietary protein/energy levels. Eur. Poult. Sci..

[bib0003] Attia Y.A., Bovera F., Wang J., Al-Harthi M.A., Kim W.K. (2020). Multiple amino acid supplementations to low-protein diets: eeffect on performance, carcass yield, meat quality and nitrogen excretion of finishing broilers under hot climate conditions. Animals.

[bib0004] Aviagen. 2019. Ross 708 performance objectives. Accessed Jan, 2023. https://aviagen.com/assets/Tech_Center/Ross_Broiler/RossxRoss708-BroilerPerformanceObjectives2019-EN.pdf.

[bib0005] Awad E.A., Najaa M., Zulaikha Z.A., Zulkifli I., Soleimani A.F. (2020). Effects of heat stress on growth performance, selected physiological and immunological parameters, caecal microflora, and meat quality in two broiler strains. Asian Australas. J. Anim. Sci..

[bib0006] Canadian Council on Animal Care (2009). CCAC guidelines on: the care and use of farm animals in research, teaching and testing.

[bib0007] Chrystal P.V., Greenhalgh S., Selle P.H., Liu S.Y. (2020). Facilitating the acceptance of tangibly reduced-crude protein diets for chicken-meat production. Anim. Nutr..

[bib0008] Eits R.M., Kwakkel R.P., Verstegen M.W.A., Stoutjesdijk P., De Greef K.H. (2002). Protein and lipid deposition rates in male broiler chickens: sseparate responses to amino acids and protein-free energy. Poult. Sci..

[bib0009] Fuller H.L., Rendon M. (1977). Energetic efficiency of different dietary fats for growth of young chicks. Poult. Sci..

[bib0010] Fuller M.F., Crofts R.M. (1977). The protein-sparing effect of carbohydrate. Br. J. Nutr..

[bib0011] Ghazalah A.A., Abd - Elsa M.O., Ali A.M. (2008). Influence of dietary energy and poultry fat on the response of broiler chicks to heat therm. Int. J. Poult. Sci..

[bib0012] Goo D., Kim J.H., Park G.H., Reyes J.B.D., Kil D.Y. (2019). Effect of heat stress and stocking density on growth performance, breast meat quality, and intestinal barrier function in broiler chickens. Animals.

[bib0013] Ji L., Laouadi A., Shu C., Gaur A., Lacasse M., Wang L. (2022). Evaluating approaches of selecting extreme hot years for assessing building overheating conditions during heatwaves. Energy Build..

[bib0014] Johnson C.A., Duong T., Latham R.E., Shirley R.B., Lee J.T. (2020). Effects of amino acid and energy density on growth performance and processing yield of mixed-sex cobb 700 × MV broiler chickens. J. Appl. Poult. Res..

[bib0015] Khoddami A., Chrystal P.V., Selle P.H., Liu S.Y. (2018). Dietary starch to lipid ratios influence growth performance, nutrient utilisation and carcass traits in broiler chickens offered diets with different energy densities. PLoS One.

[bib0016] Kyriazakis I., Emmans G.C. (1992). The effects of varying protein and energy intakes on the growth and body composition of pigs. Br. J. Nutr..

[bib0017] Lemme A. (2021). AMINOChick® 3.0 - updated amino acid recommendation tool for broilers with additional features. Aminonews®.

[bib0018] Lentfer T., Pendl H., Gebhardt-Henrich S.G., Fröhlich E.K.F., von Borell E. (2015). H/L ratio as a measurement of stress in laying hens – methodology and reliability. Br. Poult. Sci..

[bib0019] Lugata J.K., Ortega A.D., Szabó C. (2022). The role of methionine supplementation on oxidative stress and antioxidant status of poultry-A review. Agriculture.

[bib0020] Maharjan P., Martinez D.A., Weil J., Suesuttajit N., Umberson C., Mullenix G., Hilton K.M., Beitia A., Coon C.N. (2021). Review: pphysiological growth trend of current meat broilers and dietary protein and energy management approaches for sustainable broiler production. Animal.

[bib0021] Maharjan P., Mullenix G., Hilton K., Caldas J., Beitia A., Weil J., Suesuttajit N., Kalinowski A., Yacoubi N., Naranjo V., England J., Coon C. (2020). Effect of digestible amino acids to energy ratios on performance and yield of two broiler lines housed in different grow-out environmental temperatures. Poult. Sci..

[bib0022] Mateos G.G., Sell J.L., Eastwood J.A. (1982). Rate of food passage (transit time) as influenced by level of supplemental fat. Poult. Sci..

[bib0023] Morales A., Gómez T., Villalobos Y.D., Bernal H., Htoo J.K., González-Vega J.C., Espinoza S., Yáñez J., Cervantes M. (2020). Dietary protein-bound or free amino acids differently affect intestinal morphology, gene expression of amino acid transporters, and serum amino acids of pigs exposed to heat stress. Anim. Sci. J..

[bib0024] Musharaf N.A., Latshaw J.D. (1999). Heat increment as affected by protein and amino acid nutrition. Worlds Poult. Sci. J..

[bib0025] Nawab A., Ibtisham F., Li G., Kieser B., Wu J., Liu W., Zhao Y., Nawab Y., Li K., Xiao M., An L. (2018). Heat stress in poultry production: mmitigation strategies to overcome the future challenges facing the global poultry industry. J. Therm. Biol..

[bib0026] Popoola I.O., Popoola O.R., Adeyemi A.A., Ojeniyi O.M., Olaleru I.F., Oluwadele F.J., Akinwumi E.O. (2020). Overall performance, carcass yield, meat safety potentials and economic value of heat-stressed broilers fed diets with balanced electrolytes. Food Nutr. Sci..

[bib0027] Rand N.T., Scott H.M., Kummerow F.A. (1958). Dietary fat in the nutrition of the growing chick. Poult. Sci..

[bib0028] Rastogi D., Lehner F., Ashfaq M. (2020). Revisiting recent U.S. heat waves in a warmer and more humid climate. Geophys. Res. Lett..

[bib0029] Scheffé H. (1958). Experiments with mixtures. J. R. Stat. Soc. Ser. B Stat. Methodol..

[bib0030] Soares K.R., Lara L.J., Martins N.R., Silva R.R., Pereira L.F., Cardeal P.C., de Teixeira M. (2020). Protein diets for growing broilers created under a thermoneutral environment or heat stress. Anim. Feed Sci. Technol..

[bib0031] Teyssier J.R., Preynat A., Cozannet P., Briens M., Mauromoustakos A., Greene E.S., Owens C.M., Dridi S., Rochell S.J. (2022). Constant and cyclic chronic heat stress models differentially influence growth performance, carcass traits and meat quality of broilers. Poult. Sci..

[bib0032] Vogtmann H., Pfirter H.P., Prabucki A.L. (1975). A new method of determining metabolisability of energy and digestibility of fatty acids in broiler diets. Br. Poult. Sci..

[bib0033] Zaboli G., Huang X., Feng X., Ahn D.U. (2018). How can heat stress affect chicken meat quality? – a review. Poult. Sci..

[bib0034] Zhang Y.Y., Liu Y.S., Li J.L., Xing T., Jiang Y., Zhang L., Gao F. (2022). Role of dietary resistant starch in the regulation of broiler immunological characteristics. Br. J. Nutr..

